# Muscular anatomy of an entoproct creeping-type larva reveals extraordinary high complexity and potential shared characters with mollusks

**DOI:** 10.1186/s12862-015-0394-1

**Published:** 2015-07-03

**Authors:** Julia Merkel, Bernhard Lieb, Andreas Wanninger

**Affiliations:** Institute of Zoology, Johannes Gutenberg University, 55099 Mainz, Germany; Department of Integrative Zoology, University of Vienna, 1090 Vienna, Austria

**Keywords:** Tetraneuralia, Lacunifera, Spiralia, Lophotrochozoa, Kamptozoa, Evolution, Evodevo, Phylogeny, Myogenesis, Trochophore

## Abstract

**Background:**

Entoprocta (Kamptozoa) is an enigmatic, acoelomate, tentacle-bearing phylum with indirect development, either via a swimming- or a creeping-type larva and still debated phylogenetic position within Lophotrochozoa. Recent morphological and neuro-anatomical studies on the creeping-type larva support a close relationship of Entoprocta and Mollusca, with a number of shared apomorphies including a tetraneurous nervous system and a complex serotonin-expressing apical organ. However, many morphological traits of entoproct larvae, in particular of the putative basal creeping-type larva, remain elusive.

**Results:**

Applying fluorescent markers and 3D modeling, we found that this larval type has the most complex musculature hitherto described for any lophotrochozoan larva. The muscle systems identified include numerous novel and most likely creeping-type larva-specific structures such as frontal organ retractors, several other muscle fibers originating from the frontal organ, and longitudinal prototroch muscles. Interestingly, we found distinct muscle sets that are also present in several mollusks. These include paired sets of dorso-ventral muscles that intercross ventrally above the foot sole and a paired enrolling muscle that is distinct from the musculature of the body wall.

**Conclusion:**

Our data add further morphological support for an entoproct-mollusk relationship (Tetraneuralia) and strongly argue for the presence of an enrolling musculature as well as seriality (but not segmentation) in the last common tetraneuralian ancestor. The evolutionary driving forces that have led to the emergence of the extraordinarily complex muscular architecture in this short-lived, non-feeding entoproct larval type remain unknown, as are the processes that give rise to the highly different and much simpler muscular bodyplan of the adult entoproct during metamorphosis.

## Background

Entoprocta (=Kamptozoa) are microscopic, mostly marine, sessile suspension feeders. So far, approximately 150 species have been described, which are usually divided into four subgroups: the solitary Loxosomatidae and the colonial Loxocalypodidae, Barentsiidae and Pedicellinidae [1, 2]. The typical tentacle crown which surrounds both mouth and anus is part of the calyx which houses the U-shaped gut, typically one pair of protonephridia, the reproductive organs and the cerebral ganglion. Entoprocts reproduce asexually by budding or sexually via two different larval types, the supposedly basal, lecithotrophic creeping-type and the more common planktotrophic, swimming-type larva [1, 3].

The phylogenetic relationships of Entoprocta are still highly debated and today three major concepts are considered. Classical studies have argued for a close relationship of Entoprocta and Ectoprocta (“Bryozoa”-concept), mainly based on similarities during larval metamorphosis [3, 4]. Some recent molecular studies seemingly support the traditional Bryozoa-concept [5–7], although this view has repeatedly been challenged by, e.g., embryological data that demonstrated spiral cleavage in entoprocts, and thus a significant difference to the radially cleaving ectoprocts [8]. With the discovery of the Cycliophora, microscopic sessile organisms found on the mouthparts of lobsters [9], an alternative concept was established, thereby comprising cycliophorans, entoprocts and ectoprocts as a monophyletic assemblage (“Polyzoa”-concept), or - as a slight deviation from that - a sistergroup relationship solely consisting of cycliophorans and entoprocts [10, 11]. Based on a number of apomorphies shared by the entoproct creeping-type larva and larval and adult mollusks, the so-called Sinusoida- or Lacunifera-concept [12–14], which had previously suggested a monophyletic clade comprising Entoprocta and Mollusca, was recently revived, and the resulting assemblage was termed “Tetraneuralia” based on shared larval and adult neural characters [15–19]. Thereby, the creeping-type entoproct larva and the polyplacophoran larva revealed a similar architecture of the larval apical organ with numerous flask-shaped as well as peripheral cells, together with a tetraneurous condition of the longitudinal nerve cords, which so far had been considered autapomorphic for Mollusca [16, 18–21]. Even more, the creeping-type larva turned out to constitute a mosaic of larval and adult molluscan characters, featuring a distinct creeping foot with a ciliated gliding sole, pedal glands, anteriorly placed cirri and a ventrally intercrossing dorso-ventral musculature as typical traits of adult mollusks [17, 19]. All these data are particularly relevant for inferring entoproct relationships, because this complex larva is commonly considered the basal entoproct larval type and not the much simpler (and better-known) planktotrophic swimming-type larva [3]. In order to shed further light on the morphology of the still enigmatic entoproct creeping-type larva and to contribute novel data to the discussion on entoproct evolution, we studied the myoanatomy of this larva in *Loxosomella murmanica* using confocal microscopy and 3D modeling.

## Methods

### Animals and fixation

Individuals of the solitary entoproct *Loxosomella murmanica* (Nilus 1909) live epizoically on the sipunculan *Phascolion strombus* (Montagu 1804) which inhabits empty shells of the gastropod *Turritella* sp. and the scaphopod *Antalis* sp. The shells were dredged from 30 m depth from muddy and rocky bottom at Gåsö Ränna, Gullmarsfjord, close to the Kristineberg Marine Research Station, Sweden, in June 2012. The sipunculans were removed from their shells and brooding adults of *L. murmanica* were detached from its host and kept in six-well dishes until larvae were released. Larvae were relaxed with 7 % MgCl_2_ and fixed in 4 % paraformaldehyde (PFA) in 0.1 M phosphate buffered saline (PBS) for 1 h at room temperature. Afterwards, the larvae were rinsed in 0.1 M PBS (3x15min) and stored at 4 °C in 0.1 M PBS containing 0.1 % sodium azide (NaN_3_).

### Immunocytochemistry, confocal microscopy and 3D reconstruction

After storage, the larvae were rinsed (3x15min) in 0.1 M PBS and transferred into 0.1 M PBS + 0.2 % Triton X-100 (PBT) for 1 h for permeabilization. F-actin staining was carried out with a 1:20 dilution of Alexa Fluor 488 phalloidin (Invitrogen, Molecular Probes; Eugene, OR, USA) in PBT. For nucleic acid staining, 0.5 % DAPI (Invitrogen, Molecular Probes, Eugene, OR, USA) was added and the samples were incubated for 4 h in the dark. Then, larvae were rinsed in 0.1 M PBS (3x15min) and embedded on glass slides in Fluoromount G (Southern Biotech, Birmingham, AL, USA). Samples were examined using a Leica SP5 II confocal microscope. Optical sections had a step size of 0.13-0.24 μm. The confocal stacks were merged into projection images with greater focal depth. 3D reconstructions were generated with the image processing software Amira 5.4 (FEI Visualization Science Group, Hillsborow, OR, USA).

## Results

### Adult and larval gross morphology of *Loxosomella murmanica*

**Fig. 1:**
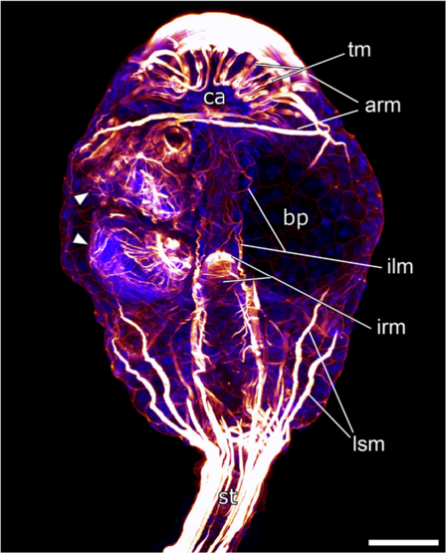
Myoanatomy of an adult *Loxosomella murmanica* specimen. Confocal micrograph with two embryos (arrowheads). Oral (i.e., ventral) facing upwards. Scale bar: 50 μm. Nucleic acid staining (blue), F-actin staining (red). arm, atrial ring muscles; bp, (empty) brood pouch; ca, calyx; ilm, intestinal longitudinal muscles; irm, intestinal ring muscles; lsm, longitudinal stalk muscles; st, stalk; tm, tentacle muscles

**Fig. 2:**
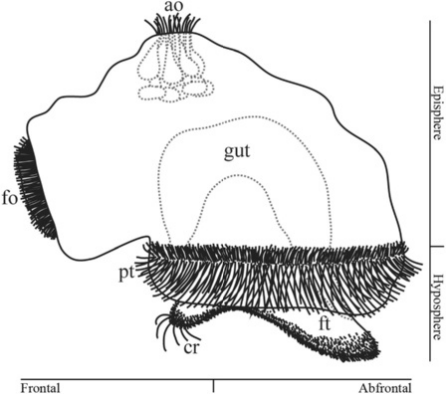
Schematic representation of a creeping-type larva of *Loxosomella murmanica*. The animal is drawn in lateral left view (after Nielsen [3], Wanninger et al. [16]). ao, apical organ; cr, frontal cirri; fo, frontal organ; ft, foot; pt, prototroch

Adult specimens of *Loxosomella murmanica* possess a flat, almost circular main body (calyx) with a crown that carries eight tentacles, a short stalk and an attachment disc, which is only slightly broader than the diameter of the stalk. The tentacle crown surrounds mouth and anus and marks the ventral (upper) side of the entoproct body [22]. In addition to the cerebral ganglion, the U-shaped digestive tract and the protonephridia [23], adults were observed to contain up to five embryos in the brood pouches of their calyces (Fig. 1). Early cleavage stages are located in the posterior-most part of the brood pouch. Later embryonic stages, close to hatching, are typically found in the uppermost part of the calyx. Released larvae are of the lecithotrophic creeping-type (Fig. 2).

The larval body is divided into an episphere and a hyposphere. The episphere comprises the region dorsal of the prototroch (pre-trochal region), including the apical organ (Fig. 2). The ventral side of the prototroch with its prominent foot sole belongs to the hyposphere (post-trochal region; see Fig. 2 and [16]). Compared with other spiralian larvae, the direction of moving is shifted by 90 degrees relative to the position of the apical organ and prototroch: while the “typical” trochophore is orientated with the apical organ forward, the larva of *Loxosomella murmanica* is oriented with the frontal organ in front during its alternating swimming and creeping movements. Therefore, the frontal organ – and not the apical organ - marks the anterior (frontal) pole of the entoproct creeping-type larva, while the opposite region is posterior (abfrontal). The gut is U-shaped and both mouth and anus reside within the hyposphere, which is framed by the prototroch and becomes the atrium in the adult entoproct (Fig. 2; see also [16]).

### Adult myoanatomy

The most prominent muscle sets of adult *Loxosomella murmanica* are formed by the atrial ring musculature, which surrounds the tentacle crown, and the longitudinal stalk musculature (arm, lsm; Fig. 1). The latter form an outer layer of muscles which run from the pedal disc into the body, where the fibers branch and form a basket-like structure towards the dorsal side of the brood pouch. The musculature of the tentacles runs in loops to the tips of the tentacles and back to the tentacle base. The musculature of the digestive tract consists of ring muscles and longitudinal muscle fibers which seem to be twisted around the gut (Fig. 1).

### Myogenesis and larval muscular anatomy in *Loxosomella murmanica*

**Fig. 3:**
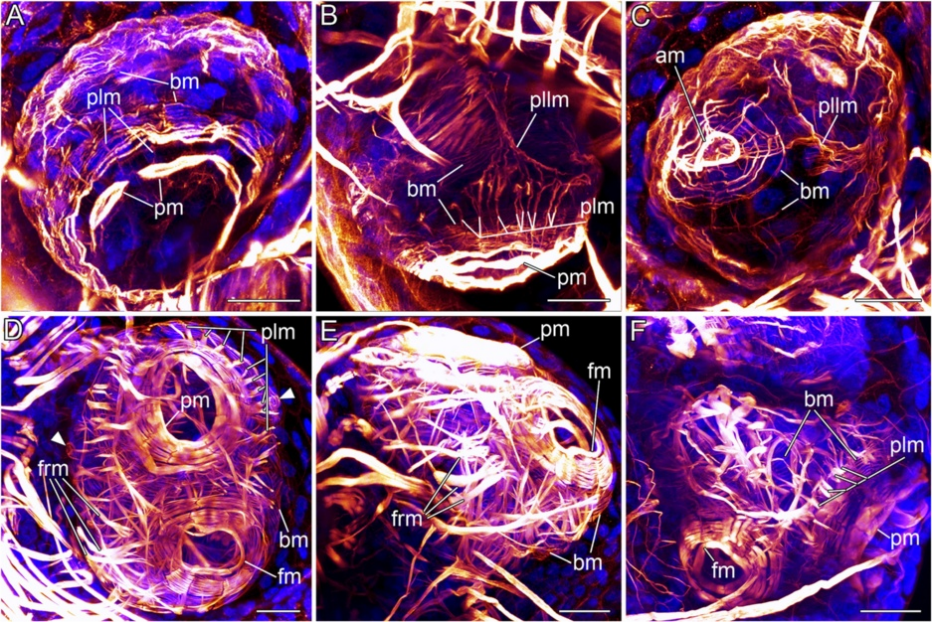
Confocal micrographs revealing the myoanatomy of different embryonic stages of *Loxosomella murmanica*. Scale bars: 20 μm. Nucleic acid staining (blue), F-actin staining (red). **a**: Ventro-lateral view of an early embryonic stage showing developing prototroch ring muscles (pm), body wall musculature (bm) and early prototroch longitudinal muscle fibers (plm). **b**: Lateral view of an early embryo. Developing body wall musculature (bm), prototroch ring muscles (pm) and prototroch longitudinal muscles (plm) are visible. The paired lateral longitudinal muscles (pllm) have formed. **c**: Apical view of an early embryo with a meshwork of concentric and longitudinal muscle fibers and developing apical ring muscles (am), body wall musculature (bm) and paired lateral longitudinal muscles (pllm). **d**: Ventral view of an older embryonic stage showing the prominent ring muscles of the prototroch (pm) and the frontal organ (fm). Prototroch longitudinal muscles (plm) and frontal organ retractor muscles (frm) have thickened. The left and right protonephridial porus with stained ring muscles (arrowheads) are visible on both sides of the embryo. **e**: Lateral view of an older embryonic stage with prominent frontal organ retractor muscles (frm). **f**: Fronto-lateral view of a late embryonic stage, probably close to hatching. The musculature resembles that of a fully developed creeping-type larva

The early embryo already exhibits a prominent prototroch ring muscle which consists of a few (four to five) muscle bundles which start to connect to neighboring muscles (pm; Fig. 3a). The body wall musculature runs in parallel to the prototroch and appears to form the precursor of the apical organ ring muscles (am, bm; Fig. 3a-c). Anlagen of prototroch longitudinal muscles are present, but are not as distinct as in older developmental stages (plm; Fig. 3a, b, d, f). Muscle fibers running from the dorso-lateral region of the early embryo seem to connect to some posteriorly positioned prototroch longitudinal muscles (Fig. 3b, c). The musculature of the apical region appears as a meshwork of concentric and longitudinal fibers (Fig. 3c).

The densely packed musculature of older embryonic stages already resembles the muscular condition of the hatched larva. The most prominent muscles are the ring muscles of the prototroch as well as those of the frontal organ and the apical organ (am, fm, pm; Fig. 3d-f). The prototroch longitudinal muscles and frontal organ retractor muscles are well developed (frm, plm; Fig. 3d). The openings of the paired protonephridia are laterally positioned (cf. [24]). The body wall ring muscles cover the entire episphere (bm; Fig. 3d-f).

### Muscular architecture of the fully developed larva

**Fig. 4:**
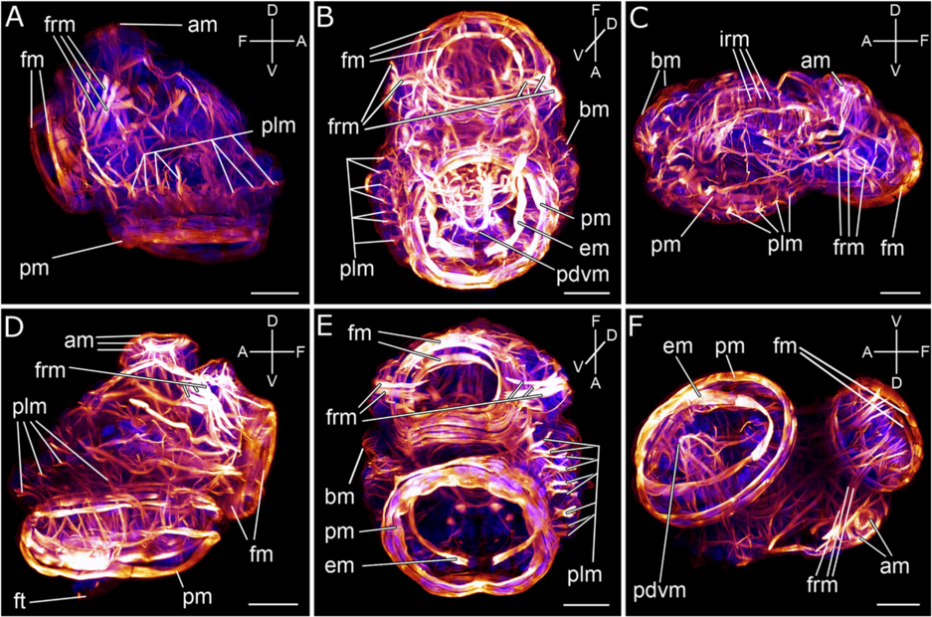
Confocal micrographs of the myoanatomy of the creeping-type larva of *Loxosomella murmanica*. Nucleic acid staining (blue), F-actin staining (red). The most prominent ring muscles are associated with the apical organ (am), frontal organ (fm) and prototroch (pm). The most prominent retractor muscles are the frontal organ retractor muscles (frm) and the prototroch longitudinal muscles (plm). **a**: Lateral view of a slightly contracted specimen. **b**: Ventral view. The pedal dorso-ventral muscles (pdvm) intercross ventrally. **c**: Dorso-lateral view of a largely expanded creeping-type larva. The ring-shaped body wall musculature (bm) as well as the ring muscles of the intestine (irm) are clearly visible. **d**: Lateral view of a slightly contracted specimen showing the posterior tip of the foot. **e**: Ventral view. The prototroch longitudinal muscles (plm) have pushed the horseshoe-shaped enrolling muscle (em) inside the larva, so that the usually more dorsally located prototroch ring muscles (pm) come to lie ventrally to the enrolling muscle (em). **f**: Ventro-lateral view of a semi-expanded creeping-type larva. Here, the horseshoe-shaped enrolling muscle (em) lies ventrally to the prototroch ring muscle (pm). The pedal dorso-ventral muscles (pdvm) intercross ventrally. a, abfrontal; d, dorsal; f, frontal; v, ventral

**Fig. 5:**
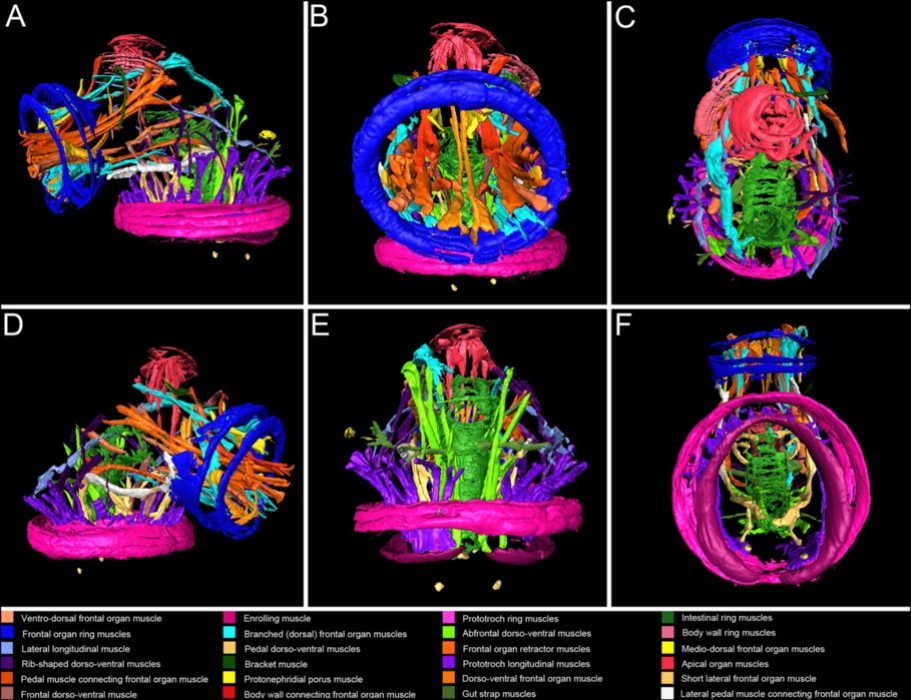
3D reconstructions of the muscular architecture of the creeping-type larva of *Loxosomella murmanica*. Reconstructions are based on the confocal microscopy dataset shown in Fig. 4 f. **a**: Fronto-lateral view. **b**: Frontal view. **c**: Dorsal view (i.e., facing the apical organ). **d**: Fronto-lateral view. **e**: Abfrontal view. **f**: Ventral view

**Fig. 6:**
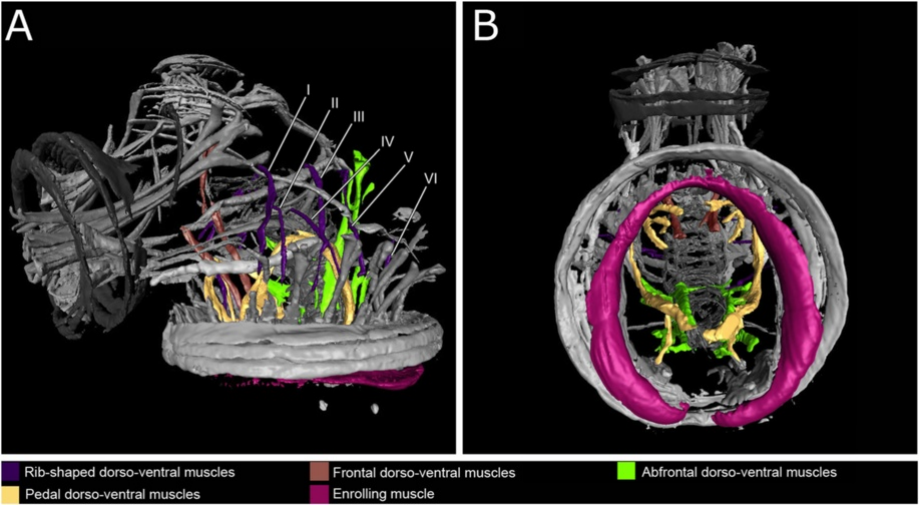
3D reconstructions of the myoanatomy of a creeping-type larva of *Loxosomella murmanica*. Reconstructions are based on the confocal microscopy dataset shown in Fig. 4f. Dorso-ventral muscle sets and enrolling muscle are highlighted. **a**: Fronto-lateral view. **b**: Ventral view

The musculature of the released entoproct creeping-type larva is very complex. A broad band of outer ring muscles runs in parallel to the prominent ring muscles of the prototroch and the apical organ (am, pm; Fig. 4a, c). Ventrally to the prototroch muscles is a horseshoe-shaped, posteriorly open muscle fiber, the enrolling muscle (em; Figs. 4b, f; 5e, f; 6a, b). Approximately 28 prototroch longitudinal muscles connect to the enrolling muscle (plm; Figs. 4; 5a, e). When contracted, the prototroch longitudinal muscles pull the enrolling muscle through the other prototroch ring muscles so that these ring muscles come to lie ventrally to the enrolling muscle (compare Fig. 4e and f). On each side of the larva, five to six lateral prototroch longitudinal muscles are connected to fine, rib-shaped dorso-ventral muscle fibers, which run close to the body wall (Figs. 5a, c, d, e; 6a, b). Six to eight abfrontal prototroch longitudinal muscles are attached to the abfrontally branched paired lateral longitudinal muscle, which curves ventrally in direction of the body wall between apical and frontal organ (Fig. 5a, d, e). A pair of pedal muscles, shaped like an inverted U and herein termed “pedal dorso-ventral muscles”, is surrounded by the prototroch muscles and the enrolling muscle and emerges close to the prototroch longitudinal muscles (Figs. 5a, d; 6a, b). The frontal part of each muscle resembles a trident (Figs. 5f; 6b). The abfrontal part is unbranched and intercrosses with the abfrontal part of the other muscle ventral to the prototroch in the posterior third of the foot (pdvm; Figs. 4b, f; 5f; 6b). Two pairs of muscles emerge from the dorsal tip of the pedal dorso-ventral muscles, the dorsal and lateral fronto-pedal muscles (Fig. 5a, d). Both pairs project towards the frontal organ ring muscle, whereas the dorsal fronto-pedal muscle is surrounded by the frontal organ ring muscles (Fig. 5a, b, d). The frontal dorso-ventral muscles are framed by the trident end of the pedal dorso-ventral muscles and seem to run towards the ring muscle system of the apical organ (Figs. 5a, d, 6a). The abfrontal dorso-ventral muscles run from both sides of the gut/anus straight towards the dorsal body wall (Figs. 5e; 6a, b). Another pair of muscles originates from the hindgut, orthogonally and in half of the length of the dorso-ventral muscles. It continues towards the lateral body wall (Fig. 5e). Muscles originating from the middle part of the gut seem to be attached to the dorsal body wall (Fig. 5a). Both muscle types originating from the gut are classified as “gut strap muscles”.

Several muscles, which project straight towards the ventral side of the larva, protrude from the ring muscles of the apical organ (Fig. 5a, b, d, e). Some of these muscles seem to be attached to the apical organ ring muscles, others may be surrounded by these ring muscles (am; Figs. 4a, c, d; 5a, b, d, e). The frontal organ ring muscle surrounds several muscles which extend far within the larval body (fm; Figs. 4d, f; 5a-d). The most eye-catching muscles are formed by three prominent muscle pairs which are located laterally and in the horizontal plane of the frontal organ, herein termed “frontal organ retractors” (frm; Figs. 4a-f; 5a, b, d). Their tips, in the most frontal region of the larva, are slightly bifurcated; their abfrontal part can be branched or simple. At least one pair is attached to a lateral, thickened part of the body wall musculature close to the apical organ (Fig. 5a, b). The other pair extends even more to the lateral sides of the larva. Another pair of branched muscles is attached to the ring-shaped body wall muscles (Fig. 5a-c). A branched muscle at the apical pole of the frontal organ, the medio-dorsal frontal organ muscle, expands towards the dorsal body wall between apical and frontal organ (Fig. 5a-d). The dorso-ventral frontal organ muscle originates at the ventral pole of the frontal organ ring muscles and curves towards the dorsal side of the larva (Fig. 5a-d). The lateral short frontal organ muscle emerges from the most lateral side of the frontal organ ring muscles and projects laterally in posterior direction (Fig. 5a, b). A relative prominent muscle pair, the longitudinal frontal organ muscle, runs in parallel to the frontal organ retractor muscles, extends further, and nearly reaches the abfronto-dorsal part of the larva (Fig. 5a-d). The highly branching dorsal frontal organ muscles originate at the apical pole of the frontal organ ring muscles and curve in parallel to the dorsal body wall towards the abfrontal side of the larva (Fig. 5a-e). Some of the frontal tips of these muscles seem to be attached to the frontal organ ring muscle. Another pair of branched muscles protrudes at the ventral side of the frontal organ ring muscles and runs in parallel to the muscles that originate from the dorsal tip of the pedal dorso-ventral muscle (Fig. 5a, b, d). A bracket-shaped unpaired muscle is positioned between the region of the apical organ and frontal organ. It lies in parallel to the rib-shaped dorso-ventral muscles mentioned above and runs close to the body wall in ventral direction (Fig. 5a, c).

## Discussion

### Comparison of the myoanatomy of entoproct larval types

Sexual reproduction in entoprocts results in the formation of one of two different larval types: the more common, but most likely derived, planktotrophic swimming-type larva or the supposedly basal, lecithotrophic, creeping-type larva [1, 3].

The body wall musculature of the swimming-type larva of *Loxosomella atkinsae* consists of ring muscles which surround the entire larval body [25]. The apical ring muscles and the ring muscles of the prototroch form the most prominent muscle sets of this larval type. Furthermore, about 40 prototroch longitudinal muscles originate from the most ventral prototroch ring muscle (“main prototroch constrictor” in [25]) and extend in direction of the body wall above the most dorsal prototroch ring muscle [25]. In the swimming-type larva, the apical organ ring muscle is connected to several longitudinal muscles: one pair of main inner longitudinal retractor muscles, one abfrontal longitudinal muscle and a pair of median muscles [25]. A pair of longitudinal muscles splits below the apical organ ring muscle, at the level of the frontal ganglion, and extends laterally to the digestive tract and establishes contact with the prototroch ring muscles. On the abfrontal side of the larva, a pair of diagonal muscles originates from the prototroch ring muscles and inserts below the apical organ ring muscles [25]. An additional short abfronto-ventral pair of muscles arises close to the abfrontal diagonal muscle but seems to terminate already at the most ventral set of the episphere body wall ring muscles. A pair of frontal diagonal muscles inserts at the prototroch constrictor muscle and terminates below the apical organ ring muscle [25].

While this brief summary shows that a number of distinct larval muscle systems are present in this relative small and simple-looking entoproct swimming-type larva, the muscular architecture of the creeping-type larva appears far more complex. Similar to the swimming-type larva, the episphere of the larval body contains numerous outer ring muscles (Fig. 4b-d). Both larval types share a prominent apical organ muscle system, consisting of several densely packed ring muscles (cf. [25] and Figs. 4d, f; 5c herein).

Differences between the swimming- and creeping-type larva are found concerning the number of the prototroch longitudinal muscles, which is considerably higher in the swimming-type larva (40 instead of 28; cf. [25] and Figs. 4; 5a, d, e herein). Probably due to a much simpler architecture of the frontal organ in the swimming-type larva of *Loxosomella atkinsae*, the number of muscle bundles originating from the frontal organ is very low compared to the creeping-type larva of *L. murmanica*. Only one pair of muscles, the paired lateral longitudinal muscle, protrudes from the level of the frontal organ of the swimming larva and splits ventrally to form contact with the prototroch ring muscles [25]. A similar muscle pair can be found in the creeping-type larva (the lateral longitudinal muscle), whereby small muscle fibers branch off to come in contact with the prototroch longitudinal muscles rather than with the prototroch ring muscles, as in the swimming larva (Fig. 5a, d, e). The dorsal end of this muscle seems to terminate not far from the frontal organ ring muscle, right below the prominent ring muscle set between apical and frontal organ (Fig. 5a). The position of the paired abfrontal diagonal muscles of the swimming-type larva between apical organ and prototroch is similar to the position of the abfrontal dorso-ventral muscles of the creeping-type larva (Fig. 5e). However, since the latter muscle pair does not contain fibers that intercross ventrally, homology of these two muscles between both larval types appears at least questionable.

Probably one of the most interesting muscle sets of the creeping-type larva, the horseshoe-shaped, posteriorly open enrolling muscle, is most likely a homolog of the main prototroch constrictor of the swimming-type larva (cf. [25]). We suggest the enrolling muscle/main prototroch constrictor muscle to be independent of the prototroch ring muscle system because (i) the position of this muscle changes by contraction of the prototroch longitudinal muscles from a ventral to dorsal position relative to the prototroch ring muscles and (ii) the shape of the enrolling muscle/main prototroch constrictor muscle is horseshoe-shaped (i.e., posteriorly open) in contrast to the prototroch muscle which forms a closed ring. The entoproct enrolling muscle may have a central function during metamorphosis, when the larva has already settled. During this process the hyposphere retracts and is enclosed through the contraction of a ring of cells above the prototroch [3, 4]. We suggest this ring of cells to be part of the enrolling muscle due to its position in the contracted larva.

### Comparison of the musculature of the entoproct creeping-type larva with that of other lophotrochozoans

Due to the highly unique overall morphology of the entoproct creeping-type larva and the high complexity of its musculature, which undoubtedly contains numerous apomorphies, comparisons to other lophotrochozoans is somewhat difficult. To our surprise, however, we found some muscle systems that bear important differences as well as similarities to that of other taxa and we focus on these in the following.

#### Body wall

Taxa with a so-called three-layered body wall musculature, consisting of an outer circular, inner longitudinal and intermediate oblique muscle layer, can be found in nearly all worm-shaped lophotrochozoan phyla, e.g., in various annelids including sipunculans and hirudineans [26–28], nemertines [29, 30], aculiferan molluscs [31–33] and in polyclad and rhabditophoran Platyhelminthes [34–36]. However, individual elements of the three-layered body wall pattern are often absent in the body wall of lophotrochozoan worms as, e.g., diagonal muscle fibers in many polychaetes [37–39]. The loss of specific muscle layers is therefore likely to be the result of secondary simplification.

Our data show that the episphere of the creeping-type larva is more or less covered with ring muscles (Figs. 3; 4), while longitudinal and oblique elements are completely missing. The adult muscle system of *Loxosomella murmanica* consists, among others, of longitudinal stalk musculature and the atrial ring muscles (Fig. 1). Muscle sets covering the complete adult body are lacking. In contrast, adult *Loxosomella vivipara* and *L. parguerensis* possess a fine outer ring muscle layer, covering parts of the body, as well as longitudinal and oblique muscle elements in the foot, stalk and calyx [40]. A true body wall musculature could not be observed in any of the investigated entoproct species, and we suggest that a lophotrochozoan-like body wall is absent in entoprocts due to secondary loss. Whether or not the adult muscles develop from larval muscle sets is still uncertain, but rather than a complete loss of the complex larval musculature, incorporation of some larval elements into the adult muscular bodyplan by remodeling seems probable. A metamorphosing creeping-type larva already shows striking similarities to an adult entoproct: the larva attaches to the substratum with the contracted frontal organ, while a ring of cells above the prototroch contracts below the retracted hyposphere [3, 4]. The larval gut rotates by 90°, larval ciliary bands disintegrate and adult cilia are formed on tentacle buds, which are exposed when the atrium reopens [3, 4]. A transformation of the frontal organ ring musculature as foot musculature and/or of the prototroch ring muscles as atrial ring musculature (Fig. 2) appears highly likely, especially when considering the relative position of these prominent muscle sets in a settled larva. Further muscle units, such as the frontal organ retractors, may contribute to the formation of the longitudinal stalk musculature, but since details on the emergence of the adult entoproct body plan from the larval one are entirely lacking, these issues require reassessment of entoproct metamorphosis using modern methods.

#### Dorso-ventral musculature

Dorso-ventral muscle fibers are part of many lophotrochozoan body plans, e.g., polychaetes [37], platyhelminths [35] and mollusks [33, 41]. Thereby, diagnostic for mollusks and entoprocts alone is the medioventral intercrossing of parts of the dorso-ventral musculature [15–17]. Recent studies have shown that the larvae of polyplacophorans and the neomeniomorph *Wirenia argentea* share a transitory seven-fold seriality of dorso-ventral muscles in their ontogeny [33]. The entoproct creeping-type larva possesses different sets of dorso-ventral muscle fibers (Figs. 5a, e, f; 6a, b). The most prominent set, the abfrontal dorso-ventral musculature, is located in the abfrontal part of the larvae, originating around the anus and running close to the hindgut, straight to the dorsal body wall (Figs. 5e; 6a, b).

Six pairs of rib-shaped muscles present another set of dorso-ventral musculature. These muscle fibers originate ventrally, surrounded by the prototroch longitudinal muscles, and bend laterally, probably inserting at the dorsal body wall (Figs. 5a; 6a, b). Additional dorso-ventral muscle pairs are found in the medio-frontal part of the larva and run towards the apical organ (Figs. 5a, b; 6a, b; frontal dorso-ventral muscles) and into the foot (Figs. 5a, d, f; 6a, b; pedal dorso-ventral muscles). These pedal dorso-ventral muscles are very strong muscle fibers, shaped like an inverted U, and seem to intercross ventrally in the posterior-third part of the animal, right above the pedal sole (Figs. 4b, f; 5a, d, f; 6a, b). These muscles are most likely the intercrossing dorso-ventral muscles previously recognized by transmission electron microscopy (see Fig. 6c in [17]).

#### Enrolling muscles

Enrolling muscles are independent lateral muscle systems that occur in some larval and adult aculiferan mollusks [33, 41]. In both entoproct larval types, a muscle system is present which bears striking similarities to the enrolling muscle of polyplacophoran and larval neomeniomorph aplacophoran mollusks: in both phyla, the enrolling muscle is independent of the body wall and extends along the entire length of the pedal sole on the ventro-lateral side (Figs. 5f; 6a, b cf. [25, 33]). In the larva of the neomeniomorph *Wirenia* as well as in the swimming- and creeping-type entoproct larvae, this enrolling muscle is horseshoe-shaped, i.e., posteriorly open (Figs. 5f; 6a, b [25, 33]). Similar to (adult) polyplacophoran mollusks, the creeping-type larva is able to contract across the entire length of the body. Due to its position above the prototroch in the contracted creeping-type larva, the entoproct enrolling muscle might have an important role during metamorphosis. While this muscle system persists in adult polyplacophorans and is remodeled to become a part of the longitudinal body wall musculature in the neomeniomorph *Wirenia*, its postmetamorphic fate in entoprocts remains unknown.

## Conclusions

Compared to other spiralian trochophore-like larvae, the muscular system of the creeping-type larva of the solitary entoproct *Loxosomella murmanica* is highly complex. Despite some muscle sets, which can be homologized between the two entoproct larval types (e.g., the prototroch longitudinal muscles, which most probably are an apomorphic character of Entoprocta), other muscle types are unique to the creeping-type larva. These are, e.g., muscles which are associated with the frontal organ, such as the frontal organ ring muscles and the frontal organ retractors. Recent studies on the myogenesis of aculiferan mollusks (neomeniomorphs and polyplacophorans) propose a common evolutionary origin of the enrolling muscle as well as a seven-fold seriality of the dorso-ventral muscles in the last common ancestor of polyplacophorans and aplacophorans [33]. The data presented herein likewise demonstrate a distinct enrolling muscle and, among others, a set of six pairs of serially arranged and one pair of ventrally intercrossing dorso-ventral muscle fibers in the entoproct creeping-type larva. In light of the Tetraneuralia hypothesis, which suggests entoproct-mollusk monophyly based on numerous morphological characters [16, 19], this strongly argues for the existence of a distinct set of enrolling muscles as well as (muscular) seriality (albeit not annelid-like segmentation) in the ur-tetraneuralian. Whether this last common ancestor of mollusks and entoprocts bore (seven) shell plates (as is assumed for the ur-aculiferan), or whether such dorsal armature constitutes an invention of Mollusca alone, remains unknown and first requires reconstruction of the last common ancestor to all mollusks before further assessments can be made.

## Abbreviations

3D: Three dimensional
a: Abfrontal
am: Apical ring muscles
ao: Apical organ
arm: Atrial ring muscles
bm: Body wall musculature
bp: Brood pouch
ca: Calyx
cr: Frontal cirri
d: Dorsal
DAPI: 4′,6-Diamidino-2-phenylindole
em: Enrolling muscle
f: Frontal
fm: Frontal organ ring muscles
fo: Frontal organ
frm: Frontal organ retractor muscles
ft: Foot
ilm: Intestinal longitudinal muscles
irm: Intestinal ring muscles
lsm: Longitudinal stalk muscles
m: Meter
MgCl_2_: Magnesium chloride
NaN_3_: Sodium azide
PBS: Phosphate buffered saline
PBT: Phosphate buffered saline with Triton X-100
pdvm: Pedal dorso-ventral muscles
PFA: Paraformaldehyde
pllm: Paired lateral longitudinal muscle
plm: Prototroch longitudinal muscles
pm: Prototroch ring muscles
pt: Prototroch
st: Stalk
tm: Tentacle muscles
μm: Micrometer
v: Ventral

## References

[1] Emschermann P (1995). Kamptozoa. Süßwasserfauna von Mitteleuropa.

[2] Nielsen C (2010). A review of the taxa of solitary entoprocts (Loxosomatidae). Zootaxa.

[3] Nielsen C (1971). Entoproct life-cycles and the entoproct/ectoproct relationship. Ophelia.

[4] Nielsen C (2001). Animal Evolution: Interrelationships of the Living Phyla.

[5] Helmkampf M, Bruchhaus I, Hausdorf B (2008). Phylogenomic analyses of lophophorates (brachiopods, phoronids and bryozoans) confirm the Lophotrochozoa concept. Proc R Soc Lond B.

[6] Hausdorf B, Helmkampf M, Nesnidal MP, Bruchhaus I (2010). Phylogenetic relationships within the lophophorate lineages (Ectoprocta, Brachiopoda and Phoronida). Mol Phylogenet Evol.

[7] Nesnidal MP, Helmkampf M, Meyer A, Witek A, Bruchhaus I, Ebersberger I, Hankeln T, Lieb B, Struck TH, Hausdorf B (2013). New phylogenomic data support the monophyly of Lophophorata and an ectoproct-phoronid clade and indicate that Polyzoa and Kryptrochozoa are caused by systematic bias. BMC Evol Biol.

[8] Merkel J, Wollesen T, Lieb B, Wanninger A (2012). Spiral cleavage and early embryology of a loxosomatid entoproct and the usefulness of spiralian apical cross patterns for phylogenetic inferences. BMC Dev Biol.

[9] Funch P, Kristensen RM (1995). Cycliophora is a new phylum with affinities to Entoprocta and Ectoprocta. Nature.

[10] Cavalier-Smith T (1998). A revised six-kingdom system of life. Biol Rev.

[11] Hejnol A, Obst M, Stamatakis A, Ott M, Rouse GW, Edgecombe GD, Martinez P, Baguñà J, Bailly X, Jondelius U, Wiens M, Müller WEG, Seaver E, Wheeler WC, Martindale MQ, Giribet G, Dunn CW (2009). Assessing the root of bilaterian animals with scalable phylogenomic methods. Proc R Soc Lond B.

[12] Bartolomaeus T (1993). Die Leibeshöhlenverhältnisse und Verwandtschaftsbeziehungen der Spiralia. Verh Dtsch Zool Ges.

[13] Bartolomaeus T (1993). Die Leibeshöhlenverhältnisse und Nephridialorgane der Bilateria - Ultrastruktur, Entwicklung und Evolution.

[14] Ax P (1999). Das System der Metazoa.

[15] Haszprunar G (2000). Is the Aplacophora monophyletic? A cladistic point of view. Amer Malac Bull.

[16] Wanninger A, Fuchs J, Haszprunar G (2007). The anatomy of the serotonergic nervous system of an entoproct creeping-type larva and its phylogenetic implications. Invertebr Biol.

[17] Haszprunar G, Wanninger A (2008). On the fine structure of the creeping larva of *Loxosomella murmanica*: additional evidence for a clade of Kamptozoa (Entoprocta) and Mollusca. Acta Zool (Stockholm).

[18] Wanninger A (2008). Comparative lophotrochozoan neurogenesis and larval neuroanatomy: recent advances from previously neglected taxa. Acta Biol Hung.

[19] Wanninger A (2009). Shaping the things to come: ontogeny of lophotrochozoan neuromuscular systems and the Tetraneuralia concept. Biol Bull.

[20] Friedrich S, Wanninger A, Brückner M, Haszprunar G (2002). Neurogenesis in the mossy chiton, *Mopalia muscosa* (Gould) (Polyplacophora): evidence against molluscan metamerism. J Morphol.

[21] Voronezhskaya EE, Tyurin SA, Nezlin LP (2002). Neuronal development in larval chiton *Ischnochiton hakodadensis* (Mollusca: Polyplacophora). J Comp Neurol.

[22] Wanninger A (2004). Myo-anatomy of juvenile and adult loxosomatid Entoprocta and the use of muscular body plans for phylogenetic inferences. J Morphol.

[23] Nielsen C. *Entoprocts.* Synopses of the British Fauna 1989, 41:1–131

[24] Nielsen C (1967). Metamorphosis of the larva of *Loxosomella murmanica* (Nilus) (Entoprocta). Ophelia.

[25] Fuchs J, Wanninger A (2008). Reconstruction of the neuromuscular system of the swimming-type larva of *Loxosomella atkinsae* (Entoprocta) as inferred by fluorescence labelling and confocal microscopy. Org Divers Evol.

[26] Wanninger A, Koop D, Bromham L, Noonan E, Degnan BM (2005). Nervous and muscle system development in *Phascolion strombus* (Sipuncula). Dev Genes Evol.

[27] Bergter A, Hunnekuhl VS, Schniederjans M, Paululat A (2007). Evolutionary aspects of pattern formation during clitellate muscle development. Evol Dev.

[28] Kristof A, Wollesen T, Maiorova AS, Wanninger A (2011). Cellular and muscular growth patterns during sipunculan development. J Exp Zool (Mol Dev Evol).

[29] Maslakova SA, von Döhren J (2009). Larval development with transitory epidermis in *Paranemertes peregrina* and other hoplonemerteans. Biol Bull.

[30] Chernyshev AV (2010). Confocal laser scanning microscopy analysis of the phalloidin-labelled musculature in nemerteans. J Nat Hist.

[31] Wanninger A, Haszprunar G (2002). Muscle development in *Antalis entalis* (Mollusca, Scaphopoda) and its significance for scaphopod relationships. J Morphol.

[32] Wanninger A, Haszprunar G (2002). Chiton myogenesis: perspectives for the development and evolution of larval and adult muscle systems in molluscs. J Morphol.

[33] Scherholz M, Redl E, Wollesen T, Todt C, Wanninger A (2013). Aplacophoran mollusks evolved from ancestors with polyplacophoran-like features. Curr Biol.

[34] Hooge MD (2001). Evolution of body-wall musculature in the Platyhelminthes (Acoelomorpha, Catenulida, Rhabditophora). J Morphol.

[35] Bolaños DM, Litvaitis MK (2009). Embryonic muscle development in direct and indirect developing marine flatworms (Platyhelminthes, Polycladida). Evol Dev.

[36] Semmler H, Wanninger A (2010). Myogenesis in two polyclad platyhelminths with indirect development, *Pseudoceros canadensis* and *Stylostomum sanjuania*. Evol Dev.

[37] Tzetlin AB, Filippova AV: Muscular system in polychaetes (Annelida). Hydrobiologia 2005, 535/536:113–126.

[38] Purschke G, Müller MCM (2006). Evolution of body wall musculature. Integr Comp Biol.

[39] Bergter A, Paululat A (2007). Pattern of body-wall muscle differentiation during embryonic development of *Enchytraeus coronatus* (Annelida: Oligochaeta; Enchytraeidae). J Morphol.

[40] Fuchs J, Bright M, Funch P, Wanninger A (2006). Immunocytochemistry of the neuromuscular systems of *Loxosomella vivipara* and *L. parguerensis* (Entoprocta: Loxosomatidae). J Morphol.

[41] Haszprunar G, Wanninger A (2000). Molluscan muscle systems in development and evolution. J Zool Syst Evol Res.

